# Soluble Dietary Fiber from Highland Barley Bran Reduces Hepatic Lipid Accumulation in Mice via Gut Microbiota Modulation

**DOI:** 10.3390/nu17243870

**Published:** 2025-12-11

**Authors:** Xuzhao Wei, Furong Lang, Huicui Liu, Shulin Wang, Tongren Wang

**Affiliations:** College of Agriculture and Animal Husbandry, Qinghai University, Xining 810016, China; 13783230355@163.com (X.W.); 18797109877@163.com (F.L.); wangsl1970@163.com (S.W.); zztfgt@163.com (T.W.)

**Keywords:** highland barley bran, soluble dietary fiber, high-fat diet, lipid-lowering effect, gut microbiota

## Abstract

Background: Obesity has emerged as a significant public health challenge largely attributed to excessive dietary fat consumption. A growing body of evidence indicates that soluble dietary fiber (SDF) can prevent high-fat-diet (HFD)-induced obesity by modulating the gut microbiota. Our previous studies have shown that SDF derived from highland barley bran exhibits favorable lipid-lowering activity in vitro, but its lipid-lowering effect in vivo remains to be elucidated. Methods: This study aimed to investigate the lipid-lowering effects of SDF from highland barley bran in HFD-fed mice based on the gut microbiota. Mice were fed an HFD, and the intervention effects of SDF on hepatic lipid metabolism and its underlying molecular mechanisms were systematically evaluated using liver lipidomics, 16S rDNA sequencing, molecular biological techniques, and fecal microbiota transplantation (FMT). Results: Liver lipidomics analysis revealed that potential lipid biomarkers responsive to barley bran-derived SDF included phosphatidylethanolamines (PE, 18:2–20:3), phosphatidylserine (PS, 18:0–18:2), and PS (18:1–22:3). Furthermore, SDF modulated the composition and structure of the gut microbiota in HFD-fed mice. Notably, SDF increased the abundance of short-chain fatty acid (SCFA)-producing bacteria, particularly *Dubosiella*, as well as elevated SCFA levels. Conclusions: The increase in SCFAs activated the hepatic AMP-activated protein kinase α (AMPK) signaling pathway, thereby ameliorating HFD-induced disturbances in lipid metabolism, reducing hepatic lipid accumulation, and lowering serum lipid concentrations.

## 1. Introduction

Obesity, defined by the World Health Organization as “abnormal or excessive fat accumulation that may impair health”, is a widespread metabolic disorder with serious implications for global public health [1,2]. Since the 1980s, the global prevalence of obesity has nearly tripled, and current trends suggest that by 2030, around 38% of adults worldwide will be classified as overweight, with an estimated 20% categorized as obese [3]. The pathogenesis of obesity is multifactorial, involving a complex interplay between intrinsic genetic predispositions and extrinsic environmental factors such as dietary intake, energy homeostasis, family history, lifestyle patterns, and psychosocial influences [4]. The primary physiological hallmark of obesity is the excessive accumulation of adipose tissue, which results from a prolonged imbalance between energy intake, metabolic processes, and energy expenditure. Chronic consumption of an HFD contributes to prolonged positive energy balance, leading to the conversion of excess energy into triglycerides and their subsequent storage in adipose tissue, thereby promoting the onset and progression of obesity [5].

Alterations in the structure and composition of the gut microbiota represent one of the key factors associated with obesity. The composition of intestinal flora in obese individuals is significantly different from that in normal-weight individuals. Compared with normal-weight individuals, the ratio of Firmicutes/Bacteroidetes (F/B) at the phylum level of intestinal microflora in obese individuals is generally increased. The study of twins by Turnbaugh et al. demonstrated that obese individuals exhibit an increase in the relative abundance of Firmicutes and a decrease in the abundance of Bacteroidetes in the intestinal microbiota, resulting in a significantly higher F/B ratio than their lean counterparts [6]. Substantial evidence indicates that modulating gut microbiota through dietary intervention to promote lipid metabolism and weight loss constitutes a promising and feasible strategy for improving metabolic health [2]. Bioactive compounds, including dietary fiber [7,8], tea polyphenols [9], and probiotic formulations [10], can mitigate the onset and progression of obesity and its related complications by regulating the intestinal microbiota.

Dietary fiber, which is not fully digestible or utilizable by the human body, contributes negligible caloric value and therefore serves as an effective strategy for improving dietary quality and reducing overall energy intake. Based on solubility, dietary fiber is categorized into SDF and insoluble dietary fiber (IDF). Compared with IDF, SDF is more readily fermented by intestinal microorganisms. It interacts with water and undergoes rapid fermentation and softening in the colon under microbial action, leading to the production of metabolites such as SCFAs, which exert beneficial physiological effects [11]. These SCFAs inhibit hepatic lipid synthesis by activating the AMPK signaling pathway, inhibiting sterol regulatory element binding protein 1c (SREBP-1c) and 3-hydroxy-3-methyl glutaryl coenzyme A reductase (HMGCR) expression, reducing fatty acid synthase (FASN) and acetyl CoA carboxylase (ACC) activity [12,13]. By activating peroxisome proliferator-activated receptor alpha (PPARα), upregulating carnitine palmitoyltransferase 1 (CPT1), and enhancing mitochondrial fatty acid β-oxidation, SCFAs promote lipid oxidative breakdown [14]. SCFAs also have the function of regulating adipose tissue by stimulating adipocytes to secrete leptin, inhibiting appetite and increasing energy consumption [15]. In addition, SCFAs enter the blood through the colonic epithelium and affect lipid, glucose and cholesterol metabolism via interactions with G protein-coupled receptors [16].

Highland barley (Qingke) is a unique cereal grain native to the Qinghai–Tibet Plateau region of China. Its bran, a major byproduct of highland barley flour processing, is rich in dietary fiber, with a content ranging from 58.7 to 65.2% dietary fiber-significantly higher than that of wheat bran (42.3%) [17]. However, the dense structure of natural bran fiber results in a low SDF proportion of only 6.1–8.3%, limiting the full release of its functional activity. In our previous work, to enhance SDF extraction yield and improve its physical, structural, and functional properties, we developed a combined approach involving solid-state fermentation using *Lactobacillus bulgaricus* and *Streptococcus thermophilus*, followed by enzymatic extraction. This treatment significantly improved the functional characteristics of SDF, including enhanced water- and oil-holding capacity, increased solubility, a more porous and loose surface morphology, enlarged specific surface area, and improved abilities to adsorb cholesterol and bile salts. Moreover, the modified SDF exhibited notable lipid-lowering effects in vitro [18]. Nevertheless, whether highland barley bran SDF exerts lipid-lowering effects in vivo and the underlying mechanisms remain unclear. To comprehensively investigate the in vivo hypolipidemic effects and mechanistic basis of SDF derived from highland barley bran, this study employs an HFD-induced obese mouse model, integrated with metabolomics, high-throughput sequencing, and molecular biology techniques. The findings aim to provide a scientific foundation and theoretical support for the development of functional products derived from highland barley bran SDF for obesity management.

## 2. Materials and Methods

### 2.1. Materials and Chemicals

The preparation of highland barley bran SDF followed the methodology described by Wei et al. [18], utilizing a mixed fermentation process with *Lactobacillus bulgaricus* and *Streptococcus thermophilus* at a ratio of 4:2 (*w*/*w*), followed by enzymatic extraction to obtain SDF with a purity of 89%. The detailed process of preparing SDF was shown in the Supplementary Figure S1.

### 2.2. Experimental Instruments

Scientific Multiskan Sky Automated Microplate Reader, Beijing Pengkun Boyuan Science and Technology Development Co., Ltd., Beijing, China; Tissuelyser-96 Tissue Homogenizer, Shanghai Jingxin Experimental Technology Co., Ltd., Shanghai, China; Analytical Balance, Langfang Zhongyi Technology Co., Ltd. Langfang, China; Eclipse Ci-L Upright Microscope-Nikon Corporation, Tokyo, Japan; SCIEX Triple Quad™ 6500+ Mass Spectrometer, SCIEX Corporation, Marlborough, MA, USA; Centrifuge, Heraeus Fresco 17, Thermo Fisher Scientific Inc., Waltham, MA, USA; CFX96TM Optics Module, CFX96TM Real-Time System, BIO-RAD Laboratories, Inc., Hercules, CA, USA; Electrophoresis System-Beijing Liuyi Biotechnology Co., Ltd., Beijing, China; Benchtop Vortex Mixer, AHN myLab^®^ VT-03, AHN Biotechnologie GmbH, Nordhausen, Germany; Microplate Reader, Multiskan FC, Thermo Fisher Scientific Inc., Waltham, MA, USA.

### 2.3. Animals and Treatments

A total of eighteen male SPF-grade C57BL/6J mice, aged five weeks and weighing approximately 15 g, were obtained from Sibefu Biotechnology Co., Ltd. (Beijing, China) (Certification No. SCXK(Jing)2024-0001). The mice were maintained in a clean-grade barrier environment at the Qinghai Institute for Endemic Disease Prevention and Control, under standardized conditions including a temperature of 25 ± 2 °C, relative humidity of 50 ± 15%, and a 12 h light-dark cycle. Following a one-week acclimatization period, the mice were randomly assigned to three experimental groups (*n* = 6 per group): the normal diet control group (ND group), the HFD group, and the SDF intervention group (SDF group), which received HFD supplemented with 0.5 g/kg body weight of SDF. The ND group received a purified diet with 10% fat-derived energy (Supplementary Table S1) and regular drinking water, while the HFD and SDF groups were fed a high-fat purified diet providing 60% fat-derived energy (Supplementary Table S1) along with regular drinking water. Following an 8-week feeding period, the mice were fasted overnight, anesthetized, and euthanized via cervical dislocation after blood collection from the orbital sinus. Blood samples were centrifuged at 3500 rpm for 15 min at 4 °C to collect serum. Liver tissues were carefully excised, washed with cold physiological saline to eliminate residual blood, and weighed accurately. Serum, liver specimens, and fecal samples were immediately frozen in liquid nitrogen and kept at −80 °C until further analysis.

### 2.4. Determination of Body Weight and Food Efficiency Ratio

The body weight and food intake of mice were recorded weekly and the food efficiency ratio was calculated according to Formula (1).

(1) $$\mathrm{Food}\;\mathrm{efficiency}\;\mathrm{ration}\;\left(\%\right)=\frac{\mathrm{Total}\;\mathrm{weight}\;\mathrm{gain}}{\mathrm{Total}\;\mathrm{food}\;\mathrm{intake}}$$

### 2.5. Assessment of Blood Lipid Levels

Assay kits supplied by Nanjing Jiancheng Bioengineering Institute (Nanjing, China) were utilized to determine the concentrations of total cholesterol (TC), total triglycerides (TG), high-density lipoprotein cholesterol (HDL-c), low-density lipoprotein cholesterol (LDL-c), alanine aminotransferase (ALT), and aspartate aminotransferase (AST) in mouse serum, with all procedures strictly following the manufacturer’s recommended guidelines.

### 2.6. Histological Examination of Liver Tissue

Mouse liver tissues were immersed in 4% paraformaldehyde at ambient temperature for 48 h, followed by dehydration via gradient ethanol solutions, xylene-mediated clearing for transparency, and subsequent embedding in paraffin wax. A microtome was employed to cut the paraffin-embedded specimens into 4-μm-thick sections, which were then subjected to hematoxylin and eosin (H&E) staining for histological examination, in accordance with the protocol described in Reference [19].

### 2.7. The Liver Index of Mice

The hepatic index of mice was calculated according to Formula (2).

(2) $$\mathrm{Liver}\;\mathrm{index}/\%=\frac{\mathrm{Liver}\;\mathrm{weight}}{\mathrm{Body}\;\mathrm{weight}}\times 100$$

### 2.8. Determination of Hepatic Tissue Lipid Content

A 50 mg portion of liver tissue was weighed and homogenized in ice-cold physiological saline, then centrifuged at 1000× *g* for 10 min. The supernatant was harvested and used to measure TG and TC concentrations according to the protocols provided with a commercial kit from Nanjing Jiancheng Bioengineering Institute (Nanjing, China) [20]. Meanwhile, 50 mg of liver tissue was homogenized in absolute ethanol and centrifuged at 1000× *g* for 10 min. The resulting supernatant was analyzed for AST and ALT concentrations using an assay kit from Nanjing Jiancheng Bioengineering Institute (Nanjing, China) [21].

### 2.9. Hepatic Lipidomics

20 mg of liver tissue was weighed on dry ice and transferred to a tube containing steel beads. Subsequently, 400 μL of water was added, and the mixture was vortexed for 60 s, followed by homogenization at 45 Hz for 4 min and ultrasonic disruption for 5 min in an ice-water bath. This procedure was repeated three times. From the resulting homogenate, 50 μL was aliquoted into a new tube, combined with 150 μL of water, and then mixed with 800 μL of extraction solvent (MTBE: MeOH = 5:1, containing internal standards). The sample was vortexed for 60 s and then sonicated for 10 min in an ice-water bath. Following this, it was centrifuged at 3000 rpm (4 °C) for 15 min, and 500 μL of the resulting supernatant was collected. The supernatant was vacuum-dried at 37 °C, and the residue was reconstituted in 150 μL of reconstitution solution (DCM: MeOH: H_2_O = 60:30:4.5). The mixture was vortexed for 30 s and ultrasonicated for 10 min in an ice-water bath. Following this, samples were centrifuged again at 12,000 rpm (4 °C) for 15 min. Finally, 70 μL of the resulting supernatant was transferred to an autosampler vial for LC-MS analysis. Furthermore, a quality control (QC) sample was created by pooling 20 μL of supernatant from each individual sample, and this pooled QC was analyzed concurrently with the experimental samples to monitor analytical consistency [22].

Chromatographic separation of the target compounds was carried out using an ACQUITY Premier ultra-high performance liquid chromatography (UHPLC) system (ACQUITY Premier, Waters, Milford, CT, USA) [23]. The mobile phase and flow rate were described in Supplementary Table S2. Detection and method development were performed using a SCIEX Triple Quad™ 6500+ mass spectrometer equipped with an electrospray ionization source. Key source parameters included an IonSpray voltage of +5500 V (positive mode) or −4500 V (negative mode), curtain gas set at 35 psi, ion source gases 1 and 2 both at 50 psi, source temperature of 350 °C, and a declustering potential of ±80 V. Data acquisition and quantification were conducted using SCIEX Analyst Software (v1.6.3) in conjunction with DATA DRIVEN FLOW (v2.0.3.11). The absolute amount of each target lipid was determined by comparing its peak area to that of the corresponding class-specific internal standard.

### 2.10. RT-qPCR

Total RNA was extracted from mouse liver tissues using a total RNA extraction kit (Service Biotechnology Co., Ltd., Wuhan, China). The total RNA was reverse transcribed using a reverse transcription kit. The mRNA expression levels in mouse liver tissues were then detected by a two-step method. The PCR program was 30 s of pre-denaturation at 95 °C, 15 s of denaturation at 95 °C, and 30 s of annealing/extension at 60 °C for 40 cycles. The relative mRNA expression of each target gene was calculated using the 2^−ΔΔCt^ method, with primer sequences provided in Supplementary Table S3.

### 2.11. Protein Extraction from Liver Tissues and Western Blot Assay

Liver tissue proteins were isolated and subjected to Western blot analysis in accordance with the protocol described in our previous research [19]. Detailed information regarding the primary antibodies used in the present study is provided in Supplementary Table S4.

### 2.12. Analysis of Gut Microbiota

One week prior to sacrifice, each mouse was individually housed in a sterile cage on a daily basis to allow for unrestricted fecal excretion. Fresh fecal samples were collected using sterile forceps and immediately transferred into 1.5 mL cryovials. Following the collection of 200 mg fresh fecal samples, total genomic DNA was extracted with a commercial DNA isolation kit (Service Biotechnology Co., Ltd., Wuhan, China), strictly in line with the manufacturer’s recommended procedures. The integrity of the extracted DNA was assessed via 1% agarose gel electrophoresis, while its purity was assessed using a microplate reader. Samples displaying distinct electrophoretic bands and intact genomic profiles, fulfilling the criteria for subsequent sequencing, had their concentrations quantified with a NanoDrop spectrophotometer. The V3–V4 hypervariable regions of the bacterial 16S rDNA were amplified via PCR using the primer pair 341F (CCTACGGGNGGCWGCAG) and 805R (GACTACHVGGGTATCTAATCC). The resulting PCR amplicons were verified by agarose gel electrophoresis and subsequently purified with the Qiagen Gel Extraction Kit (75510-019, Invitrogen, Carlsbad, CA, USA). High-throughput sequencing was carried out on the Illumina NovaSeq 6000 platform at Shanghai Baiqu Biomedical Technology Co., Ltd. (Shanghai, China), Raw sequence data were processed and analyzed using the bioinformatics pipeline detailed in our earlier studies [24].

### 2.13. Determination of SCFAs in the Fecal Samples of Mice

Fecal samples were placed into 2 mL EP tubes and resuspended in 1 mL of ultrapure water, followed by brief vortex mixing (10 s). Steel beads were introduced, and the samples were mechanically lysed at 40 Hz for 4 min. After lysis, the suspensions underwent three cycles of ultrasonication on ice (4 °C, 5 min per cycle). The homogenates were then centrifuged at 5000 rpm and 4 °C for 20 min. A 0.8 mL aliquot of the supernatant from each sample was transferred into a new 2 mL tube. Thereafter, 0.1 mL of 50% sulfuric acid (H_2_SO_4_) and 0.8 mL of extraction solvent (methyl tert-butyl ether supplemented with 25 mg/L 2-methylvaleric acid as the internal standard) were added sequentially. The resultant mixture was vortexed for 10 s, oscillated for 10 min, and subjected to additional sonication on ice (4 °C, 10 min). Following incubation at −20 °C for 30 min to facilitate phase separation, the samples were centrifuged again at 10,000 rpm and 4 °C for 15 min. The upper organic layer was collected and transferred to a GC-MS vial, which was then prepared for subsequent detection and analysis.

A SHIMADZU GC2030-QP2020 NX system fitted with a capillary column (HP-FFAP, Agilent Technologies, Inc., Palo Alto, CA, USA) was employed for GC-MS analysis. The instrument parameter settings were shown in Supplementary Table S5.

### 2.14. FMT

A total of eighteen male SPF-grade C57BL/6J donor mice, aged five weeks, were obtained from Sibefu Biotechnology Co., Ltd. (Beijing, China). The mice were maintained in a clean-grade barrier environment at the Qinghai Institute for Endemic Disease Prevention and Control, under standardized conditions including a temperature of 25 ± 2 °C, relative humidity of 50 ± 15%, and a 12 h light-dark cycle. Following a one-week acclimatization period, the mice were randomly assigned to three groups (*n* = 6 per group): ND, HFD and SDF group (received HFD supplemented with 0.5 g/kg body weight of SDF). The ND group received a purified diet with 10% fat-derived energy and regular drinking water, while the HFD and SDF groups were fed a high-fat purified diet providing 60% fat-derived energy along with regular drinking water. After four weeks of feeding, daily collection of fresh fecal samples was conducted from the ND, HFD, and SDF groups. 200 mg of fresh feces were suspended in 2 mL sterile saline solution. The solution was vigorously vortexed for 10 s using a benchtop vortex mixer, followed by centrifugation at 800× *g* for 3 min. The supernatant was collected as the transplantation material. To minimize changes in microbial composition, the fecal transplant suspension was freshly prepared within 10 min prior to oral administration.

A total of eighteen male SPF-grade C57BL/6J recipient mice, aged five weeks, were obtained from Sibefu (Beijing) Biotechnology Co., Ltd. (Beijing, China), and were maintained in a clean-grade barrier environment at the Qinghai Institute for Endemic Disease Prevention and Control, under standardized conditions including a temperature of 25 ± 2 °C, relative humidity of 50 ± 15%, and a 12 h light-dark cycle. After a one-week acclimatization period, the mice were administered a 200 μL cocktail of antibiotics (ampicillin 1 g/L, metronidazole 1 g/L, vancomycin 0.5 g/L, and neomycin 0.5 g/L) daily for two weeks to deplete the native gut microbiota. Following antibiotic pretreatment, the mice were randomly assigned to three groups. They were the FND (ND group-derived fecal microbiota), FHFD (HFD group-derived fecal microbiota), and FSDF (SDF group-derived fecal microbiota) groups, respectively. The recipient mice were fed a high-fat purified diet providing 60% fat-derived energy along with regular drinking water. Each group (*n* = 6) received daily gavages of 100 μL of freshly prepared fecal suspension from donor mice of the ND, HFD, or SDF groups, respectively [25,26]. To reduce the differences among the donor microbial communities, equal amounts of feces from each donor mouse were collected and mixed together to prepare a bacterial suspension for FMT. The transplanted mice were housed in groups of four per cage. The study concluded after four weeks, at which point serum, liver tissue, fecal samples, and other specimens were collected and stored at −80 °C for further analysis.

### 2.15. Statistical Analyses

Data are expressed as mean ± standard deviation. Statistical analysis was performed using SPSS 26 software (IBM, Armonk, NY, USA), with the Tukey–Kramer method applied to determine significant differences, where *p* ≤ 0.05 was deemed statistically significant. Data analysis was performed using GraphPad Prism version 10.1.2 software (GraphPad, San Diego, CA, USA).

## 3. Results

### 3.1. Effects of SDF on Body Weight and Lipid Levels in Mice Fed an HFD

**Figure 1:**
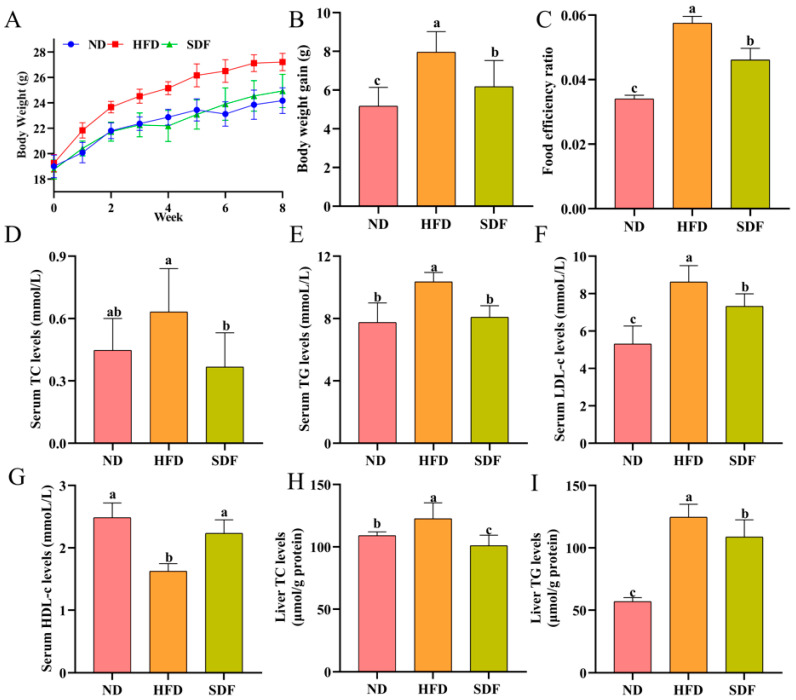
Effects of SDF on body weight and lipid levels in mice fed an HFD (*n* = 6). (**A**) The change trend of body weight in mice. (**B**) The gain of body weight. (**C**) Food efficiency ratio. (**D**) TC levels in serum. (**E**) TG levels in serum. (**F**) LDL-c levels in serum. (**G**) HDL-c levels in serum. (**H**) Liver TC levels. (**I**) Liver TG levels. Bars labeled with different letters (a, b, c) denote statistically significant differences at *p* ≤ 0.05, as determined by the Tukey–Kramer method.

The impacts of SDF on body weight and blood lipid levels in HFD mice were illustrated in Figure 1. Mice in the HFD group showed a significant increase in body weight gain compared to those in the ND group, whereas SDF intervention markedly attenuated this increase (Figure 1A,B). Additionally, the food efficiency ratio was significantly higher in the HFD group than in the SDF group (*p* < 0.05; Figure 1C), indicating that SDF may suppress HFD-induced weight gain by reducing the food efficiency ratio. As shown in Figure 1D–G, compared to the ND group, the HFD group showed a significant elevation in serum TG and LDL-c levels (*p* < 0.05) and a marked reduction in HDL-c levels (*p* < 0.05). Although TC levels were higher in the HFD group, the difference did not reach statistical significance (*p* > 0.05). In contrast, SDF supplementation significantly reduced serum levels of TC, TG, and LDL-c (*p* < 0.05) and significantly increased HDL-c levels (*p* < 0.05) compared to the HFD group, demonstrating that SDF effectively ameliorates HFD-induced serum lipid dysregulation. In parallel, compared with the ND group, the levels of TC and TG in liver tissues of the HFD group were significantly elevated (*p* < 0.05), indicating that HFD may induce hepatic lipid accumulation. Following SDF intervention, the TC and TG levels in liver tissues of the SDF group were significantly reduced (*p* < 0.05), suggesting that SDF can mitigate HFD-induced hepatic lipid deposition and improve associated disturbances in hepatic lipid metabolism (Figure 1H,I). In summary, SDF not only attenuates HFD-induced weight gain by reducing the food efficiency ratio, but also effectively modulates serum lipid profiles and reduces hepatic lipid accumulation, thereby ameliorating HFD-induced dysregulation of lipid metabolism.

### 3.2. Effects of SDF on Hepatic Lipid Accumulation and Hepatic Function

**Figure 2:**
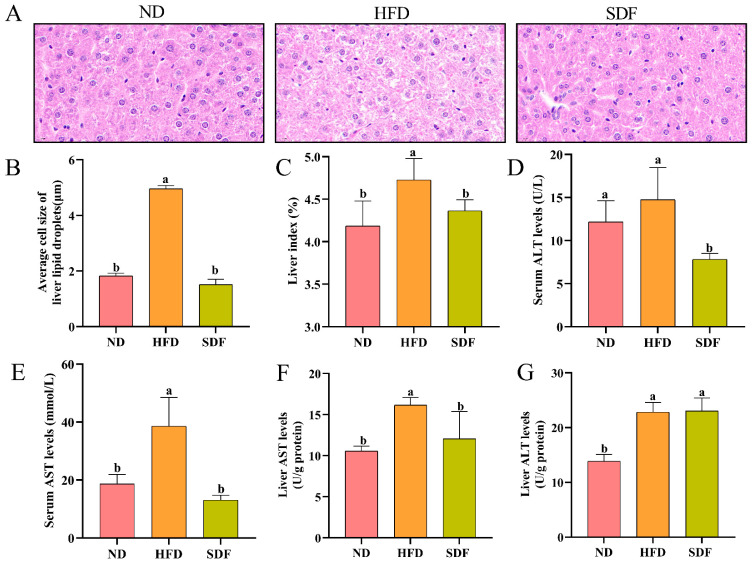
Impact of SDF on hepatic steatosis and hepatic function in HFD mice (n = 6). (**A**) H&E staining of liver tissue (Scale: 20 μm). (**B**) Liver lipid droplet size. (**C**) Hepatic index. (**D**) Serum ALT concentration. (**E**) Serum AST concentration. (**F**) Hepatic AST levels. (**G**)Hepatic ALT levels. Bars labeled with different letters (a, b) denote statistically significant differences at *p* ≤ 0.05, as determined by the Tukey–Kramer method.

An HFD can induce excessive lipid accumulation in hepatocytes, leading to hepatic steatosis and impaired liver function [27]. The effects of SDF on hepatic lipid accumulation and liver function in HFD-fed mice were presented in Figure 2. As shown in Figure 2A,B, liver tissues from the HFD group exhibited significant lipid vacuolation, cellular swelling, and disruption of normal cellular architecture. Intracellular lipid droplets were extensively aggregated, with an average diameter of 4.96 ± 0.12 μm, significantly larger than that observed in the ND group (1.82 ± 0.11 μm, *p* < 0.05). In contrast, SDF intervention substantially reduced hepatic lipid vacuolation and significantly decreased lipid droplet size compared to the HFD group (*p* < 0.05), indicating that SDF alleviates HFD-induced hepatic lipid accumulation and associated histopathological alterations. The liver index in the HFD group was 4.73%, significantly higher than that in the ND group (4.16%, *p* < 0.05). Following SDF intervention, the liver index was markedly reduced relative to the HFD group (*p* < 0.05), further supporting the protective effect of SDF against HFD-induced hepatic injury. ALT and AST serve as critical biomarkers for assessing hepatic function. As illustrated in Figure 2D,E, although serum ALT activity was elevated in the HFD group, no statistically significant difference was observed compared to the ND group (*p* > 0.05); however, serum AST activity was significantly increased (*p* < 0.05). Following intervention with SDF, both serum ALT and AST activities were significantly reduced compared to the HFD group (*p* < 0.05). Moreover, hepatic tissue ALT and AST activities in the HFD group were significantly elevated relative to the ND group (*p* < 0.05). After SDF administration, hepatic AST activity was markedly lower than that of the HFD group (Figure 2F,G, *p* < 0.05). These results demonstrate that highland barley bran SDF effectively ameliorates HFD-induced liver dysfunction.

### 3.3. SDF Modulates Hepatic Lipid Metabolism Dysregulation in HFD Mice

**Figure 3:**
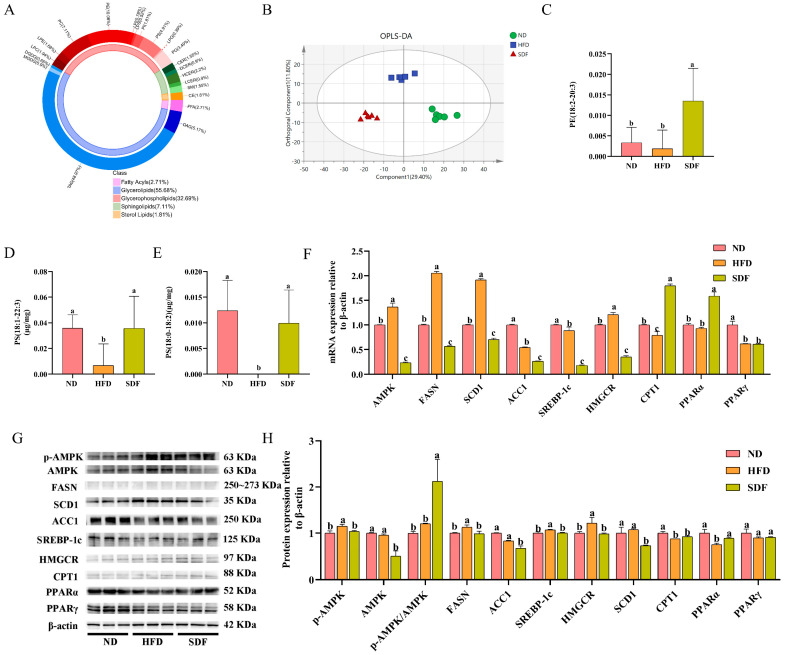
Impact of SDF on Hepatic Lipid Metabolism Disorder in HFD-Fed Mice (*n* = 6). (**A**) Liver lipid composition. (**B**) Orthogonal partial least squares discriminant analysis (OPLS-DA). (**C**–**E**) Potential lipid markers in response to SDF. (**F**) Lipid metabolism-related gene expression in liver tissues. (**G**) Western Blot band of liver lipid metabolism protein. (**H**) Gray value of each band. Bars labeled with different letters (a, b, c) denote statistically significant differences at *p* ≤ 0.05, as determined by the Tukey–Kramer method. Adenosine 5′-monophosphate (AMP)-activated protein kinase (AMPK), Phosphorylated AMPK (p-AMPK), Fatty Acid Synthase (FASN), Stearoyl-Coenzyme A Desaturase-1 (SCD1), Acetyl CoA Carboxylase 1 (ACC1), Sterol Regulatory Element Binding Protein 1c (SREBP-1c), 3-Hydroxy-3-Methyl Glutaryl Coenzyme A Reductase (HMGCR), Carnitine Palmitoyl Transferase-1 (CPT1), Peroxisome Proliferator-activated Receptor alpha (PPARα), Peroxisome Proliferator-activated Receptor γ (PPARγ).

To evaluate the effects of SDF derived from highland barley bran on hepatic lipid composition in mice fed an HFD and to identify potential lipid biomarkers associated with liver lipid metabolism disorders, a comprehensive liver lipidomics analysis was conducted. The results were presented in Figure 3. As illustrated in Figure 3A, the hepatic lipid profile in mice is predominantly composed of triacylglycerols (TAG, 48.97%), PE (10.08%), phosphatidylcholines (PC, 7.11%), diacylglycerols (DAG, 5.17%), and PS (4.91%), among others. OPLS-DA score plots (Figure 3B) revealed clear separation between the HFD group and both the ND group and the SDF intervention group, indicating that SDF significantly modulates hepatic lipid metabolic profiles in HFD-induced mice. Based on the OPLS-DA results, differentially altered lipid metabolites were screened using the criteria of VIP > 2.5 and Fold Change > 2 or < 0.5. As illustrated in Figure 3C–E, compared with the ND group, the HFD group exhibited a significant reduction the levels of PS (18:0–18:2) and PS (18:1–22:3) (*p* < 0.05). Following SDF intervention, the hepatic proportion of PE (18:2–20:3) as well as the levels of PS (18:0–18:2) and PS (18:1–22:3) were significantly increased (*p* < 0.05). These findings suggest that PE (18:2–20:3), PS (18:0–18:2), and PS (18:1–22:3) may serve as potential biomarkers for the amelioration of hepatic lipid metabolism induced by SDF derived from highland barley bran.

Meanwhile, as illustrated in Figure 3F, compared with the ND group, HFD feeding significantly upregulated the mRNA expression of AMPK, FASN, SCD1, and HMGCR in mouse liver tissues, while markedly downregulating the mRNA expression of ACC1, SREBP-1c, CPT1, and PPARγ (*p* < 0.05). In contrast to the HFD group, intervention with SDF from highland barley bran resulted in significant downregulation of AMPK, FASN, SCD1, ACC1, SREBP-1c, and HMGCR mRNA expression, along with significant upregulation of CPT1 and PPARα mRNA expression in liver tissues (*p* < 0.05).

As shown in Figure 3G,H, HFD feeding significantly increased the protein expression of p-AMPK, FASN, SREBP-1c, and HMGCR, while decreasing the levels of ACC1, CPT1, and PPARα in liver tissue compared to the ND group (*p* < 0.05). Relative to the HFD group, SDF intervention significantly enhanced protein expression of p-AMPK and PPARα (*p* < 0.05). Moreover, SDF intervention significantly reduced the protein levels of p-AMPK, AMPK, FASN, SCD1, ACC1, SREBP-1c, and HMGCR relative to the HFD group. As a central regulator in the AMPK signaling pathway, AMPK modulates downstream proteins involved in lipid synthesis and oxidation through signal transduction. These results indicate that SDF suppresses hepatic lipid synthesis by promoting AMPK phosphorylation and suppressing the activity of key lipogenic enzymes, including FASN, SREBP-1c, SCD1, ACC1, and HMGCR. Furthermore, SDF upregulates PPARα to promote fatty acid oxidation, thereby ameliorating HFD-induced hepatic lipid metabolic disturbances.

### 3.4. SDF Modulates the Gut Microbiota and Restores SCFAs in HFD-Affected Mice

**Figure 4:**
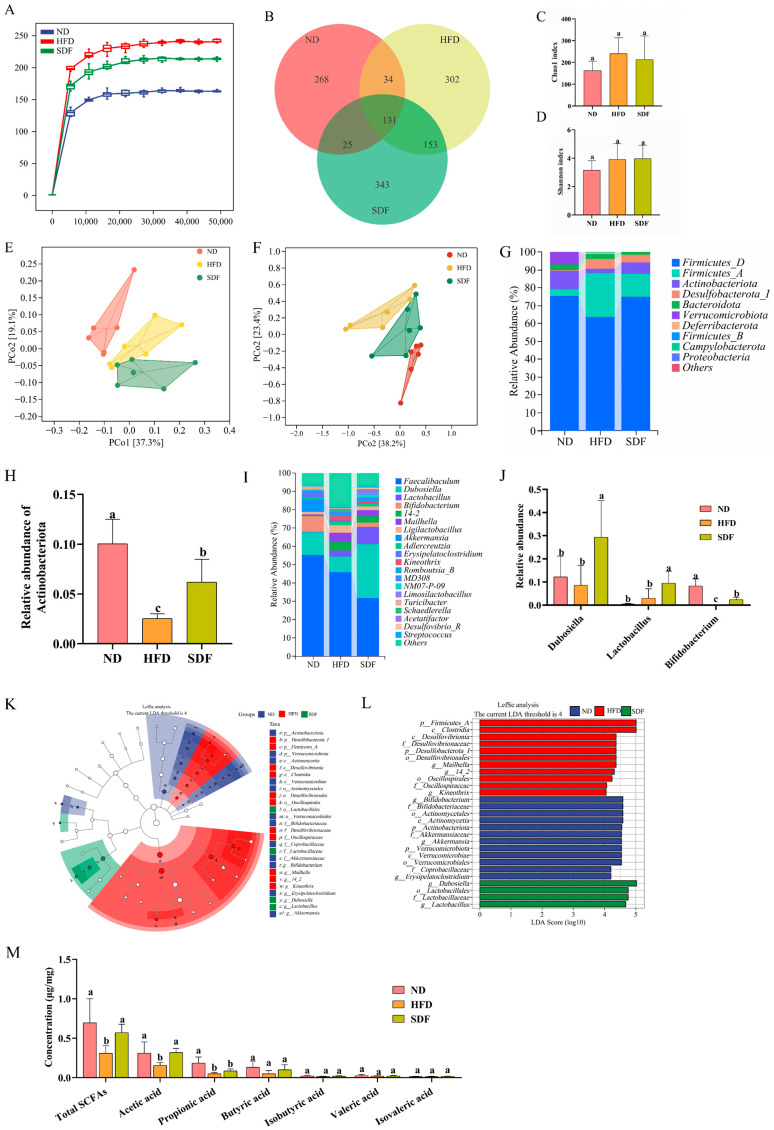
SDF Modulates the Composition and Structural Diversity of the Gut Microbiota (*n* = 6). (**A**) Rarefaction curve. (**B**) Venn diagram. (**C**) Chao1 index. (**D**) Shannon index. (**E**) Bray–Curtis based PCoA plots. (**F**) OPLS-DA analysis. (**G**) Phylum-level bacterial distribution histogram. (**H**) Actinobacteria relative abundance. (**I**) Histogram of bacterial distribution at the genus level. (**J**) Relative abundance of gut microbiota at the genus level. (**K**) Bacterial taxon phylogenetic relationship cladogram. (**L**) Distribution histogram based on linear discriminant analysis (LDA). (**M**) SCFAs level in feces of mice. Bars labeled with different letters (a, b, c) denote statistically significant differences at *p* ≤ 0.05, as determined by the Tukey–Kramer method.

Rarefaction curves indicate that the current sequencing depth has sufficiently captured the majority of gut microbial diversity in each sample (Figure 4A). The Venn diagram illustrated that a total of 913 operational taxonomic units (OTUs) were identified across the ND, HFD, and SDF groups, with varying numbers of OTUs observed in each group (Figure 4B). No significant differences in the Chao1 index and Shannon index were observed among the ND, HFD, and SDF groups (Figure 4C,D). Furthermore, Bray–Curtis based principal coordinates analysis (PCoA) and OPLS-DA analysis demonstrated strong intra-group consistency (Figure 4E,F). These findings indicate that SDF intervention significantly alters the overall structure of the gut microbiota in mice.

The relative abundances of the intestinal microbiota at the phylum and genus levels were compared across the three sample groups. At the phylum level, Firmicutes, Actinobacteriota, Desulfobacterota, Bacteroidota and Verrucomicrobiota were identified as the five most dominant phyla (Figure 4G). A further comparison of these major phyla revealed that the relative abundance of Actinobacteriota in the HFD group was significantly lower than that in the ND group, whereas it was significantly higher in the SDF group compared to the HFD group (Figure 4H, *p* < 0.05). At the genus level, *Faecalibaculum*, *Dubosiella*, *Lactobacillus*, *Bifidobacterium*, and *14-2* were the predominant genera observed in the ND, HFD, and SDF groups (Figure 4I). Noticeably, compared with the ND group, the relative abundance of *Bifidobacterium* was significantly reduced in the HFD group. In contrast, the relative abundances of *Dubosiella*, *Lactobacillus*, and *Bifidobacterium* were significantly increased in the SDF group relative to the HFD group (Figure 4J, *p* < 0.05).

To further identify the key bacterial phyla and genera contributing to structural differences in the gut microbiota across the three sample groups, LEfSe discriminant analysis (LDA score > 4, *p* < 0.05, Kruskal–Wallis test; top 30 most abundant taxa) was conducted to characterize differentially enriched microbial communities at both the phylum and genus levels. The results demonstrated that, at the phylum level, Firmicutes_A, Desulfobacterota, and Actinobacteriota were significantly associated with the observed structural variations in the gut microbiota among the three mouse groups (Figure 4K,L). At the genus level, eight genera exhibited significant differences in relative abundance. Among them, there were 3 in the ND group (*Bifidobacterium*, *Akkermansia*, and *Erysipelatoclostridium*), 3 in the HFD group (*Mailhella*, 14-2, and Kineothrix), and 2 in the SDF group (*Dubosiella* and *Lactobacillus*). These results suggest that SDF intervention can partially reshape the gut microbiota community structure of mice.

SCFAs are microbial metabolites produced through the fermentation process of indigestible dietary fibers and other complex carbohydrates mediated by gut microbiota. Our study investigates the effects of SDF on intestinal microbial activity by quantifying SCFA concentrations in fecal samples collected from mice. Relative to the ND group, the concentrations of total SCFAs, acetic acid, and propionic acid in fecal samples of HFD-fed mice were significantly decreased. In contrast, SDF intervention notably elevated the levels of total SCFAs and acetic acid (Figure 4M).

### 3.5. SDF-Mediated FMT Alleviates HFD-Induced Gut Microbiota Dysbiosis

**Figure 5:**
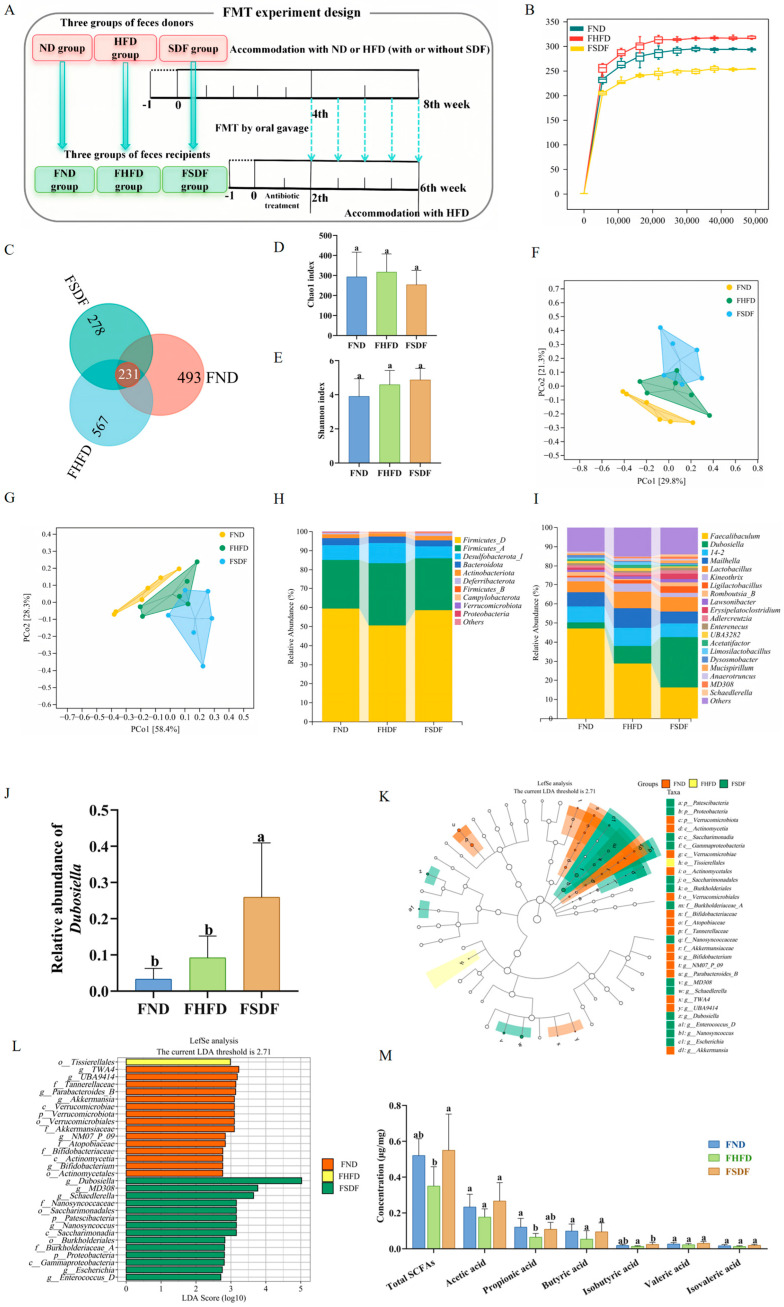
FMT of SDF mice attenuated HFD-induced damage of gut microbiota (n = 6). (**A**) FMT experimental design, FND: ND group-derived fecal microbiota; FHFD: HFD group-derived fecal microbiota; FSDF: SDF group-derived fecal microbiota. (**B**) Rarefaction curve. (**C**) Venn diagram. (**D**) Chao1 index. (**E**) Shannon index. (**F**) Bray–Curtis based PCoA plots. (**G**) PCA analysis. (**H**) Histogram of bacterial distribution at the phylum level. (**I**) Genus-level bacterial distribution histogram. (**J**) *Dubosiella* relative abundance. (**K**) Phylogenetic cladogram of bacterial taxa. (**L**) Distribution histogram based on LDA (>2.71). (**M**) SCFAs. Bars labeled with different letters (a, b) denote statistically significant differences at *p* ≤ 0.05, as determined by the Tukey–Kramer method.

To investigate the role of gut microbiota in mediating the metabolic benefits of SDF, fecal microbiota from SDF-treated donor mice was transferred into recipient mice fed an HFD, followed by evaluation of key parameters related to lipid metabolism (Figure 5A). As shown in Figure 5B, similar to the donor group, rarefaction curves indicate that the current sequencing depth has sufficiently captured the majority of gut microbial diversity in each sample. A Venn diagram revealed a total of 1338 OTUs, with distinct numbers of OTUs observed across the FND, FHFD, and FSDF groups, indicating distinct microbial community compositions among these groups (Figure 5C). Similarly, in line with the donor group, no significant differences (*p* > 0.05) in α-diversity indices (Chao1, Shannon) were observed among the FND, FHFD, and FSDF groups (Figure 5D,E). Subsequently, Bray–Curtis based PCoA and OPLS-DA analysis demonstrated strong intra-group consistency (Figure 5F,G), supporting the reliability of the sequencing data. Collectively, these analyses reveal significant inter-group differences, indicating a marked alteration in the overall composition of the intestinal microbiota following FMT.

As presented in Figure 5H, consistent with the donor group, the gut microbiota of transplanted mice comprised 10 phyla. Among these, Firmicutes, Desulfobacterota, Bacteroidota, and Actinobacteriota were recognized as the most dominant phyla. At the genus level, *Faecalibaculum*, *Dubosiella*, 14-2, *Mailhella*, and *Lactobacillus* were the predominant genera in the FND, FHFD, and FSDF groups (Figure 5I). Noticeably, compared with the FND and FHFD group, the relative abundance of *Dubosiella* was significantly increased in the FSDF group (Figure 5J, *p* < 0.05).

To further identify the key bacterial phyla and genera contributing to structural differences in the gut microbiota across the FND, FHFD, and FSDF groups, LEfSe discriminant analysis (LDA score > 2.71, *p* < 0.05, Kruskal–Wallis test; top 30 most abundant taxa) was conducted to characterize differentially enriched microbial communities at both the phylum and genus levels (Figure 5K,L). The results demonstrated that, at the phylum level, Verrucomicrobiales, Proteobacteria, and Patescibacteria were significantly associated with the observed structural variations in the gut microbiota among the three mouse groups. At the genus level, twelve genera exhibited significant differences in relative abundance. Among them, there were 6 in the FND group (*Bifidobacterium*, *NM07_P-09*, *Parabacteroides-B*, *TWA4*, *UBA9414*, *Akkermansia*), and 6 in the FSDF group (*MD308*, *Schaedlerella*, *Dubosiella*, *Enterococcus-D*, *Nanosyncoccus*, *Escherichia*). These results suggest that SDF intervention can partially reshape the gut microbiota community structure of mice. Consequently, these data suggested that FMT exerted a significant effect on changing the gut microbiota structure of HFD-fed mice at both the phylum and genus taxonomic levels.

### 3.6. Metabolic Parameters of Mice in FMT Groups

**Figure 6:**
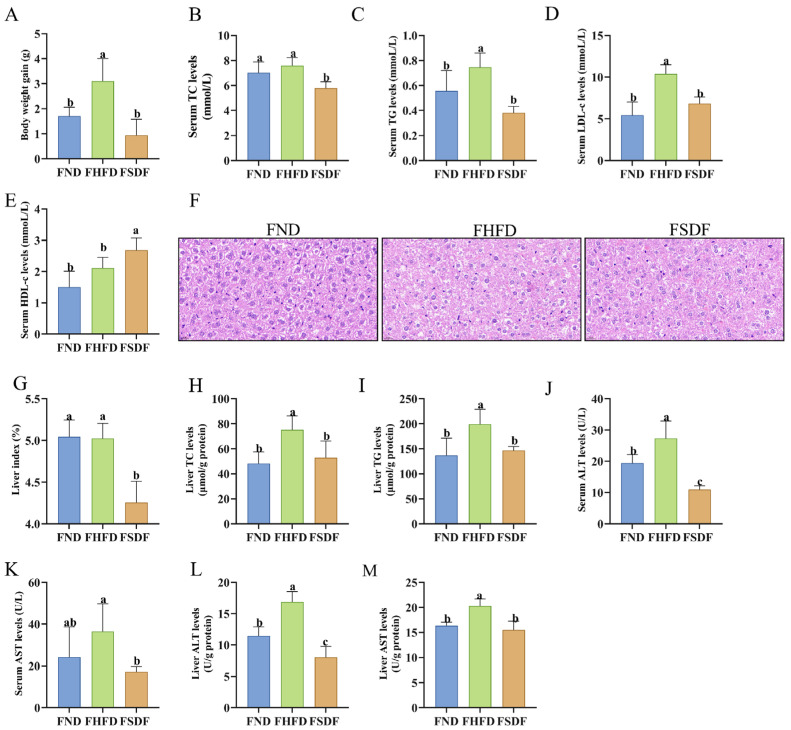
Metabolic Parameters of Mice in FMT Groups (n = 6). (**A**) Body weight gain. (**B**) Levels of TC in serum. (**C**) Levels of TG in serum. (**D**) Levels of LDL-c in serum. (**E**) Levels of HDL-c in serum. (**F**) Liver tissue H&E staining (Scale: 20 μm). (**G**) Liver index. (**H**) Hepatic TC levels. (**I**) Hepatic TG levels. (**J**) Serum ALT concentration. (**K**) Serum AST concentration. (**L**) Hepatic ALT concentration. (**M**) Hepatic AST concentration. Bars labeled with different letters (a, b, c) denote statistically significant differences at *p* ≤ 0.05, as determined by the Tukey–Kramer method.

Mice in the FND group gained 1.71 ± 0.36 g, while those in the FSDF group gained only 0.94 ± 0.64 g, both significantly less than the 3.11 ± 0.90 g observed in the FHFD group (Figure 6A, *p* < 0.05). After FMT, the body weight trajectories in the FND, FHFD, and FSDF groups closely mirrored those of their corresponding donor groups (ND, HFD, and SDF, respectively), indicating that FMT partially recapitulated the protective effect against weight gain in HFD-fed recipients. By four weeks post-transplantation, although serum TC and HDL-c levels in the FHFD group were somewhat elevated compared to the FND group, these differences were not statistically significant (Figure 6B,E; *p* > 0.05). In contrast, TG and LDL-c levels were markedly higher in the FHFD group. Importantly, the FSDF group exhibited significantly lower levels of TC, TG, and LDL-c, along with a significant increase in HDL-c, relative to the FHFD group (Figure 6B–E, *p* < 0.05). These findings demonstrate that SDF-induced alterations in gut microbiota play a crucial role in improving lipid metabolism in HFD-fed mice.

Meanwhile, H&E staining of the liver revealed an increased presence of white lipid droplets in the hepatic tissue of the FHFD group compared to the FND group, indicating lipid accumulation within hepatocytes (Figure 6F). Concurrently, the liver index was significantly elevated in the FHFD group relative to the FND group. The accumulation of lipids in hepatocytes leads to hepatic metabolic dysregulation, hepatic steatosis, and impaired liver function. In the FSDF group, both hepatic lipid droplets and the liver index were markedly reduced (Figure 6F,G), exhibiting a hepatic condition similar to that of the FN group. Furthermore, compared to the FND group, hepatic TC and TG levels in the FHFD group exhibited a significant increase (*p* < 0.05, Figure 6H,I), indicating pronounced lipid droplet accumulation in the liver. After FMT, the FSDF group showed a marked reduction in hepatic TC and TG content relative to the FHFD group (*p* < 0.05). These findings suggest that SDF ameliorates hepatic lipid accumulation in HFD-fed mice by modulating the gut microbiota. Additionally, both serum and hepatic AST and ALT levels were significantly lower in the FSDF group compared to the FHFD group (Figure 6J–M, *p* < 0.05), indicating that SDF-induced alterations in the gut microbiota improve liver function in mice subjected to an HFD.

## 4. Discussion

This study demonstrates that soluble dietary fiber (SDF) ameliorates HFD-induced hepatic lipid metabolic disorders by activating the SCFA–pAMPK signaling pathway, thereby significantly reducing serum and hepatic lipid levels and improving liver function.

Liver lipidomics analysis was performed to investigate alterations in hepatic lipid metabolites in HFD-fed mice following intervention with SDF derived from highland barley bran. Three potential lipid biomarkers responsive to SDF treatment were identified: PE (18:2–20:3), PS (18:0–18:2), and PS (18:1–22:3). PE is closely associated with liver cell regeneration. Carril et al. reported that an increased hepatic PE content promoted liver tissue regeneration in a mouse model of non-alcoholic steatohepatitis [28]. In this study, HFD feeding led to significant lipid accumulation, extensive lipid droplet deposition, and hepatocellular injury. Following SDF intervention, the number of lipid droplets in mouse liver tissue was decreased, and hepatocytes exhibited morphological recovery toward normal status, which may be linked to an elevated proportion of PE in the liver tissue. PS has been shown to act as a PPARα agonist, activating PPARα signaling and promoting fatty acid oxidation [29]. Therefore, SDF from highland barley bran increased the hepatic levels of PS (18:0–18:2) and PS (18:1–22:3) in HFD-fed mice, which was accompanied by upregulated PPARα expression and activation of CPT1, leading to enhanced lipid metabolism.

A well-balanced gut microbial community is essential for the prevention and management of obesity. The mechanisms by which gut microbiota modulation ameliorates obesity include maintaining intestinal barrier integrity and regulating key microbial metabolites, particularly SCFAs. SCFAs can enter the systemic circulation and directly or indirectly activate AMPK in hepatocytes [30,31]. For instance, propionic acid activates the AMPK signaling pathway, suppresses the expression of key lipogenic regulators such as HMGCR and SREBP-1c, and reduces the enzymatic activities of FASN and ACC, thereby inhibiting hepatic lipid synthesis [12,13]. Meanwhile, butyrate activates PPARα, leading to the upregulation of CPT1, enhanced mitochondrial fatty acid β-oxidation, and increased lipid oxidative breakdown [14]. Thus, the gut microbiota can indirectly regulate hepatic lipid metabolism through the SCFA-AMPK signaling axis. In this study, supplementation with SDF from highland barley bran significantly increased the abundance of beneficial bacteria, including Actinobacteriota, *Dubosiella*, *Lactobacillus*, and *Bifidobacterium*. Actinobacteriota have been reported to exert beneficial effects on host health [32]. *Bifidobacterium* is a key probiotic genus within the phylum Actinobacteria. Previous studies have shown that dietary supplementation with *Bifidobacterium* in HFD-fed C57BL/6J mice improves glucose tolerance and insulin sensitivity, and enhances acetic acid production [33]. These SCFAs are essential intestinal metabolites that confer beneficial effects on host health, including reduction of body weight in obese mice, amelioration of glucose and lipid metabolism disorders, and alleviation of hepatic steatosis [32]. Consistent with prior findings—such as those showing hazelnut SDF ameliorates hyperlipidemia and obesity in rats by enriching *Lactobacillus* and *Roseburia* [34], and bamboo shoot dietary fiber increasing *Bifidobacterium* and *Dubosiella* abundance in obese mice [32]—our results demonstrate that highland barley bran SDF significantly elevates fecal concentrations of total SCFAs and acetic acid in HFD-fed mice. Notably, SDF intervention significantly upregulated hepatic protein expression levels of pAMPK and total AMPK (*p* < 0.05), indicating enhanced activation of pAMPK in the liver. Furthermore, SDF downregulated the protein expression of key lipogenic genes, including HMGCR, SREBP-1c, FAS, ACC, and SCD1. Concurrently, SDF upregulated the expression of CPT1α and PPARα—critical regulators of fatty acid transport into mitochondria and subsequent β-oxidation—thereby promoting hepatic lipid catabolism [35]. Supporting evidence from Zhou et al. also indicates that bamboo shoot dietary fiber alleviates obesity by modulating host PPAR signaling and fatty acid metabolism pathways [32]. Based on these findings, we propose that SDF alleviates HFD-induced lipid metabolism disorders by enriching beneficial gut bacteria, boosting SCFA production, and consequently activating the hepatic AMPK signaling pathway.

To further confirm the mediating role of the gut microbiota in the ability of SDF from highland barley bran to ameliorate hepatic lipid metabolism disorders in HFD-fed mice, we conducted an FMT experiment. The results indicated that FMT effectively reshaped the structure and composition of the gut microbiota in recipient mice, with the microbial profile being similar to that of the donor group.

At the genus level, the relative abundance of *Dubosiella* was higher in the FSDF group compared to the FHFD group (*p* < 0.05), consistent with the elevated *Dubosiella* levels observed in the original SDF donor group relative to the HFD control group. Notably, the concentration of total SCFAs in the fecal samples of mice in the FSDF group was significantly increased. These findings collectively confirm that SDF improves hepatic lipid metabolism in HFD-fed mice by reshaping HFD-induced gut microbiota dysbiosis, elevating the relative abundance of beneficial gut bacteria (notably *Dubosiella*), thereby promoting SCFA biosynthesis and activating the SCFAs-AMPK signaling pathway. This study provides direct evidence for the causal role of gut microbiota in mediating the metabolic benefits of highland barley bran SDF.

This study still has many limitations. SCFAs have been established as important regulatory factors that act as signaling molecules via G-protein-coupled receptors, including free fatty acid receptor 2 (FFAR2) and FFAR3. However, this study did not investigate the impact of SDF on FFAR2 and FFAR3. In future studies, we will explore whether SCFA-mediated receptor signaling promotes AMPK activation and lipid metabolism. Additionally, although this study verified the mediating role of gut microbiota in the intervention of obesity by SDF through FMT, and clarified that highland barley bran SDF improved lipid metabolism disorders caused by an HFD by reshaping gut microbiota and activating the SCFAs-AMPK pathway. However, due to species differences, dietary doses, and the complexity of intestinal and liver signal transduction, the lipid-lowering mechanism of SDF needs to be further verified in clinical and cell experiments.

## 5. Conclusions

In summary, the findings of this study provide new evidence supporting the lipid-lowering effects of highland barley bran SDF in HFD mice. Briefly, intervention with highland barley bran SDF modulated the composition of the gut microbiota. Notably, SDF significantly increased the levels of SCFAs by enhancing the abundance of SCFA-producing bacteria, particularly *Dubosiella*. The elevated SCFA levels subsequently activated the AMPK signaling pathway, thereby ameliorating hepatic lipid metabolic disorders in HFD mice.

## Data Availability

The original contributions presented in this study are included in the article/Supplementary Material. Further inquiries can be directed to the corresponding author.

## Supplementary Materials

The following supporting information can be downloaded at: https://www.mdpi.com/article/10.3390/nu17243870/s1. Figure S1: The process of preparing SDF. Table S1: Diet composition. Table S2: The instrument settings of the UHPLC system. Table S3: Primer sequence. Table S4: Western blot primary antibody information. Table S5: The instrument settings of the GC-MS system.
