# Supplementary material for: Soluble Dietary Fiber from Highland Barley Bran Reduces Hepatic Lipid Accumulation in Mice via Gut Microbiota Modulation
Source: Nutrients. 2025 Dec 11;17(24):3870. doi: 10.3390/nu17243870 (PMC12736037; doi:10.3390/nu17243870)
Supplement: Supplementary file 1 [file nutrients-17-03870-s001.zip › nutrients-3993052-supplementary.pdf]

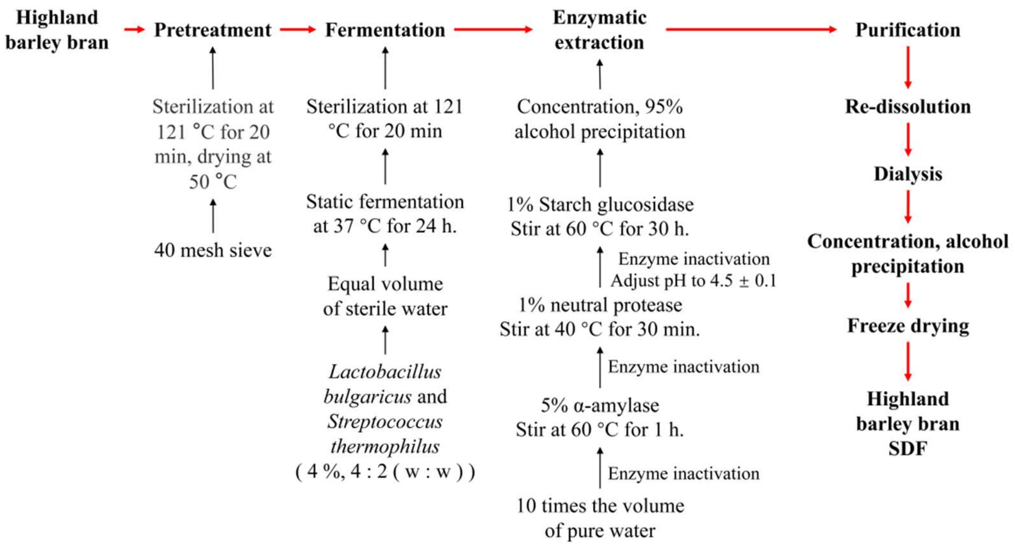

Figure S1 The process of preparing SDF

Table S1 Diet composition

Diet	Composition	Mass ratio g %	Energy ratio kcal %
Normal Diet	Protein	19.2	20
	Carbohydrates	67.3	70
	Fat	4.3	10
	Total		100
	kcal/g	3.85	
High Fat Diet	Protein	26	20
	Carbohydrates	26	20
	Fat	35	60
	Total		100
	kcal/g	5.24	

Table S2 The intrument settings of UHPLC system

Items	Parameters	
Mobile phase	Solvent A	containing 40% water and 60% acetonitrile with 10 mmol/L ammonium acetate and 0.1% acetic acid
	Solvent B	consisting of 10% acetonitrile and 90% isopropanol supplemented with the 10 mmol/L ammonium acetate and 0.1% acetic acid
Flow rate of mobile phase	0.3 mL/min	
Column temperature	45 ℃	
Sample tray temperature	10 ℃	
Injection volume	2 μL	

Table S3 Primer sequence

Gene	Primer	Sequences（5′ → 3′）
β-actin	Forward	GTGACGTTGACATCCGTAAAGA
	Reverse	GCCGGACTCATCGTACTCC
AMPK	Forward	GGGAAAGTGAAGGTGGGCAA
	Reverse	ATCTCCCGGCGGATTTTTCC
FASN	Forward	GACTCGGCTACTGACACGAC
	Reverse	CGAGTTGAGCTGGGTTAGGG
SCD1	Forward	TTCTTGCGATACACTCTGGTGC
	Reverse	CGGGATTGAATGTTCTTGTCGT
ACC1	Forward	CTTCCTGACAAACGAGTCTGG
	Reverse	CTGCCGAAACATCTCTGGGA
SREBP-1c	Forward	CCTAGTCCGAAGCCGGGTG
	Reverse	GAGCTGGAGCATGTCTTCGAT
HMGCR	Forward	ACGTGGTGTGTCTATTCGCC
	Reverse	AGCAAGCTCCCATCACCAAG
CPT1	Forward	CTATGCGCTACTCGCTGAAGG
	Reverse	GGCTTTCGACCCGAGAAGA
PPARα	Forward	AACATCGAGTGTCGAATATGTGG
	Reverse	CCGAATAGTTCGCCGAAAGAA
PPARγ	Forward	GGAAGACCACTCGCATTCCTT
	Reverse	GTAATCAGCAACCATTGGGTCA

Table S4 Western blot primary antibody information

Primary antibody	Brand and Item Number	Dilution ratio
p-AMPK	Wanleibio（WL05103）	1:1000
AMPK	Wanleibio（WL02254）	1:1000
FASN	Servicebio（GB15546）	1:1000
SCD1	Servicebio（GB113844）	1:1000
SREBP-1c	BOSTER（A00282）	1:1000
ACC1	Abways（CY5575）	1:1000
HMGCR	Servicebio（GB111352）	1:1000
CPT1	Servicebio（GB112537）	1:1000
PPARα	BOSTER（A00600）	1:1000
PPARγ	Servicebio（GB12164）	1:1000

Table S5 The intrument settings of GC-MS system

Items	Parameters
Sample volume	1 μL
Front Inlet Mode	Split Mode(5:1)
Front Inlet Septum Purge Flow	3mL/ min
Carrier Gas	Helium
Column	HP-FFAP（30m×250μm×0.25μm）
Oven Temperature Ramp	50°C hold on 1 min; raised to 150°C at a rate of 50°C<br>min−1, hold on 1 min; raised to 170°C at a rate of 10°C<br>min−1, hold on 0min;raised to 225°C at a rate of 25°C<br>min−1, hold on 1 min; raised to 240°C at a rate of 40°C<br>min−1, hold on 1min
Front Injection Temperature	220°C
Transfer Line Temperature	240°C
Ion Source Temperature	240°C
Quad Temperature	150°C
Electron Energy	-70eV
Solvent Delay	3.60 min
